# Acoustic Velocity Log Numerical Simulation and Saturation Estimation of Gas Hydrate Reservoir in Shenhu Area, South China Sea

**DOI:** 10.1155/2013/101459

**Published:** 2013-07-01

**Authors:** Kun Xiao, Changchun Zou, Biao Xiang, Jieqiong Liu

**Affiliations:** ^1^School of Geophysics and Information Technology, China University of Geosciences, Beijing 10083, China; ^2^Key Laboratory of Geo-detection (China University of Geosciences, Beijing), Ministry of Education, Beijing 100083, China

## Abstract

Gas hydrate model and free gas model are established, and two-phase theory (TPT) for numerical simulation of elastic wave velocity is adopted to investigate the unconsolidated deep-water sedimentary strata in Shenhu area, South China Sea. The relationships between compression wave (P wave) velocity and gas hydrate saturation, free gas saturation, and sediment porosity at site SH2 are studied, respectively, and gas hydrate saturation of research area is estimated by gas hydrate model. In depth of 50 to 245 m below seafloor (mbsf), as sediment porosity decreases, P wave velocity increases gradually; as gas hydrate saturation increases, P wave velocity increases gradually; as free gas saturation increases, P wave velocity decreases. This rule is almost consistent with the previous research result. In depth of 195 to 220 mbsf, the actual measurement of P wave velocity increases significantly relative to the P wave velocity of saturated water modeling, and this layer is determined to be rich in gas hydrate. The average value of gas hydrate saturation estimated from the TPT model is 23.2%, and the maximum saturation is 31.5%, which is basically in accordance with simplified three-phase equation (STPE), effective medium theory (EMT), resistivity log (Rt), and chloride anomaly method.

## 1. Introduction

Gas hydrate mainly exists in the seafloor and polar permafrost [1], and it owns the cage structure of solid crystal, which is formed by water molecules and natural gas (usually dominated by methane). The formation of gas hydrate needs a low-temperature and high-pressure environment, and the concentration of methane must exceed its solubility in the pore water. So gas hydrate is commonly distributed in the water depth greater than 300 m in the continental slope belt [2, 3]. The submarine gas hydrate reserves mainly depend on the distribution of gas hydrate area, the thickness of gas hydrate stability zone, the porosity of sedimentary layer, the saturation of gas hydrate, and so on. However, the accurate estimation of gas hydrate reserves is very difficult due to the lack of research on the determination of gas hydrate distribution and gas hydrate saturation. As gas hydrate is rich in methane, it is associated with a series of scientific issues [4], including the global carbon cycle [5], global temperature changes [6], the sea-level rise [7], and future energy supply [8]. Therefore, researches on determining gas hydrate distribution and estimating gas hydrate saturation have become the focus of the scientists all over the world. In May 2007, the gas hydrate samples and various log data of gas hydrate zone were firstly obtained in Shenhu area, South China Sea, which made a significant breakthrough in exploration of gas hydrate in China. Meanwhile, it provided a great convenience for investigating the properties of the gas hydrate reservoir [9].

Geophysical logging is an important tool for evaluating gas hydrate saturation, and valuable information can be obtained by studying the resistivity and acoustic velocity log data. According to the log data in Shenhu area, South China Sea, Chinese scholars have utilized some theoretical models and empirical formulas to estimate gas hydrate saturation [10–14], and the results of these researches have greatly promoted the process of studying gas hydrate saturation by using log data in China. However, previous studies mainly focused on the estimation of the gas hydrate saturation, and the discrepancy of the estimation of gas hydrate saturation was large because of the application of various methods. Besides, previous studies did not systematically study the relationship between any two of the gas hydrate saturation, elastic wave velocity, and sediment porosity, which led to incomplete understanding of the log data.

Foreign scholars have carried out researches on the evaluation of the marine gas hydrate saturation earlier. A variety of theoretical models or experimental models have been proposed to estimate gas hydrate saturation, such as Wyllie et al. [15] time average equation with the seismic velocity [16–18], the effective medium theory [19–23], Biot-Gassmann theory model [24–27], compression wave (P wave) velocity of thermal-elastic theory [28], the three-phase equation (TPE) [29–34], and velocity model theory based on the two-phase theory (TPT) model [35]. Moreover, Tinivella et al. [36] made a research to compare the TPT model with the TPE model for evaluating gas hydrate saturations in marine sediments, and the comparison showed that the two theoretical approaches were in very good agreement. Based on this, the TPT model has been applied to verify the TPE model and estimate the gas hydrate and free gas saturations in several different areas [37–39].

In this work, based on the log data at site SH2 in Shenhu Area, we first establish gas hydrate model and free gas model by applying elastic wave velocity numerical model of the TPT method, then study the dependence of the P wave velocity on gas hydrate saturation, free gas saturation, and sediment porosity, and finally choose the gas hydrate model to estimate gas hydrate saturation at site SH2.

## 2. Numerical Simulation of Elastic Wave Velocity by the TPT Model

The TPT model [41, 42], which supposes that the rock solid part is composed of rock matrix and gas hydrate and that the rock pore fluid is composed of free gas and water, can be used to study the elastic wave velocity model about the elastic characteristics of marine sand-shale reservoirs. Based on this theory and using the reported in Tinivella [35], the velocity relation between P wave velocity (*V*_p_) and shear wave (S wave) velocity (*V*_s_) is as follows:

(1) Vp={[(1Cm+43μ)    +(ϕeff/k)(ρm/ρf)+(1−β−2(ϕeff/k))(1−β)(1−ϕeff−β)Cb+ϕeff Cf]   ·1ρm(1−(ϕeff/k)(ρf/ρm))}1/2,Vs={μ{ρm[1−(ϕeff·ρf)/(k·ρm)]}}1/2.

Where *ϕ*_eff_ is the effective porosity, *μ* is the average rigidity of the skeleton, *ρ*_m_ is the average density, *ρ*_f_ is the density of the fluid phase, *β* is the proportionality coefficient, *k* is the coupling factor, and *C*_b_, *C*_f_ and *C*_m_ are the compressibility of the solid phase, the fluid phase, and the matrix, respectively.

## 3. Geological Setting

**Figure 1:**
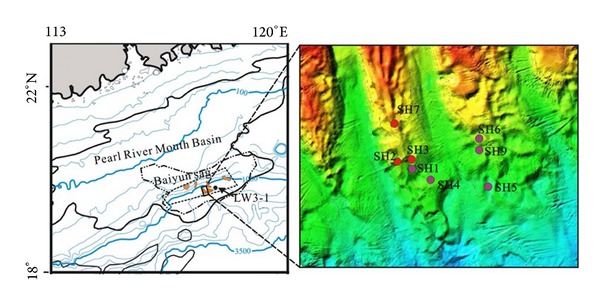
Areas of gas hydrate exploration and drilling area with drilling sites in the northern part of the South China Sea [40]. (Red dots, gas hydrate samples obtained; dark purple dots, no gas hydrate samples obtained.)

Shenhu area is considered as one of the occurrences of gas hydrate, which is located in the middle of the northern slope of the South China Sea, between the Xisha Trough and the Dongsha Islands and Baiyun Sag of Zhu II depression of the Pearl River Mouth Basin (Figure 1). Shenhu area experienced a geological evolution process similar to the northern margin of the South China Sea and eventually formed the regional sedimentary sequences in which marine sediments were the dominant composition [43]. Taking the Cenozoic sedimentary strata in the Shenhu area, for example, it is about 1000–7000 m, and the organic matter content is 0.46%–1.9% [44–46], which can provide the material base for gas hydrate. In recent years, some studies have confirmed that mud volcanoes, seafloor slips, mud diapers and other special structural units beneficial to the formation of gas hydrate are widely being developed in Shenhu area [47], and the bottom simulating reflections (BSRs) have also been identified using various geophysical methods in the northern South China Sea by Guangzhou Marine Geological Survey, China Geological Survey [48]. 

**Figure 2:**
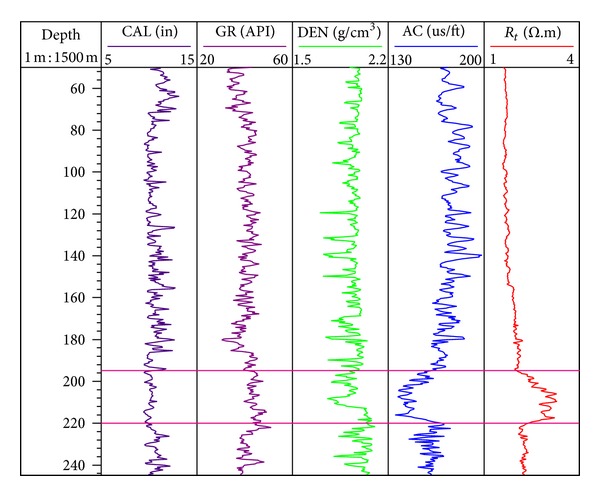
The conventional logs in site SH2 [10]. (The area delineated by a pink line is the occurrence of gas hydrate reservoir, and the depth range is 195 to 220 mbsf.)

During April–June 2007, eight sites were drilled in Shenhu area (see Figure 2), among which, sites of SH2, SH3, and SH7 in water depth of 1105 to 1423 m were determined to contain gas hydrate in recovered core samples. The thickness of gas hydrate stability zone was about 10 to 25 m [9, 49], and the sediment lithology in and above the zone was silt and silty clay respectively, according to the core data.

## 4. Data Set Used in the Study

As previous researchers have done much work on site SH2 [10–14, 40, 50], many valuable references can be used to obtain better results and verify the reliability of our research, so we select site SH2 as the research object. The water depth of site SH2 is 1232 m, and the maximum drilling depth is 245 mbsf. Figure 2 shows the conventional logs of site SH2. The measurement depth range is from 50 to 245 mbsf, and the measurement projects include caliper, density, natural gamma ray, acoustic, and resistivity logging. The occurrence of gas hydrate reservoir in this site appears to be “response characteristics of two high and two low” in the log curve, namely high resistivity, high natural gamma ray and low density, low acoustic time, especially for resistivity and acoustic logs. Besides, when the layer of well diameter changes, caliper curve can be used as the effective parameter to identify gas hydrate reservoir because the abnormality of other logs has nothing to do with the well condition.

Based on the previous research methods for the thickness of gas hydrate stability zone [51–53] and combined with the analysis of conventional log data, the gas hydrate stability zone of site SH2 is determined to be at the depth of 195 to 220 mbsf [40].

## 5. Methodology

Seafloor sediments containing gas hydrate are generally composed of rock grain, gas hydrate, water, and natural gas. In order to research the characteristics of gas hydrate reservoir, gas hydrate model and free gas model have been established in this section, and based on these two models, the numerical simulation method and the TPT are used to study the dependence of the elastic wave velocity on sediment porosity, gas hydrate saturation and free gas saturation.

### 5.1. Gas Hydrate Model

#### 5.1.1. Establishment of Gas Hydrate Model

The gas hydrate model assumes that the sediments are composed of rock grain, gas hydrate and water, and gas hydrate, is in the pore space, which is regarded as a part of the rock matrix. Supposing that *ϕ*_s_, *ϕ*_h_, *ϕ*_w_, and *ϕ*_g_ represent the volume percentage of rock grain, gas hydrate, water, and free gas in the sediments respectively, the gas hydrate model can be expressed as

(2) $$\begin{array}{c} \phi_{\text{s}}+\phi_{\text{h}}+\phi_{\text{w}}=1, \end{array}$$

(3) $$\begin{array}{c} \phi =\phi_{\text{h}}+\phi_{\text{w}}, \end{array}$$

where *ϕ* is sediment porosity.

Gas hydrate saturation (*S*_h_) and water saturation (*S*_w_) can be written as

(4) $$\begin{array}{c} S_{\text{h}}=\frac{\phi_{\text{h}}}{\phi },\\ S_{\text{w}}=\frac{\phi_{\text{w}}}{\phi }. \end{array}$$

The volume percentages of rock grain in solid phase (*S*_s_′) and gas hydrate in the solid phase (*S*_h_′) can be written, respectively, as

(5) $$\begin{array}{c} S_{\text{s}}^{\prime }=\frac{\phi_{\text{s}}}{\left(\phi_{\text{s}}+\phi_{\text{h}}\right)}, \end{array}$$

(6) $$\begin{array}{c} S_{\text{h}}^{\prime }=\frac{\phi_{\text{h}}}{\left(\phi_{\text{s}}+\phi_{\text{h}}\right)}. \end{array}$$

#### 5.1.2. The Parameter Determination for Numerical Simulation of Gas Hydrate Model Based on the TPT

In order to apply the TPT to gas hydrate model, some parameters in (1) should be known. The parameters can be determined by Tinivella and Schon's derivation formula [35, 54].

 (1) Effective porosity (*ϕ*_eff_) can be written as
  (7) $$\begin{array}{c} \phi_{\text{eff}}=\left(1-{\text{S}}_{\text{h}}\right)\phi . \end{array}$$
 (2) Average density of sediments (*ρ*_m_), density of the solid phase (*ρ*_b_), and density of the fluid phase (*ρ*_f_) can be written as
  (8) $$\begin{array}{c} \rho_{\text{m}}=\left(1-\phi_{\text{eff}}\right)\rho_{\text{b}}+\phi_{\text{eff}}\rho_{\text{f}}, \end{array}$$
  (9) $$\begin{array}{c} \rho_{\text{b}}=S_{\text{s}}^{\prime }\rho_{\text{s}}+S_{\text{h}}^{\prime }\rho_{\text{h}}, \end{array}$$
  (10) $$\begin{array}{c} \rho_{\text{f}}=\rho_{\text{w}}, \end{array}$$
  where *ρ*_s_ is density of the rock grain, *ρ*_h_ is gas hydrate density and *ρ*_w_ is water density.
  (3) Assume that the solid compressibility lies between the Voigt and Reuss averages [54]. *C*_b_ can be written as
  (11) Cb=12(Ss′Cs+Sh′Ch)+12(Ss′Cs+Sh′Ch)−1,
  where *C*_s_ is rock grain compressibility and *C*_h_ is gas hydrate compressibility.
 (4) Compressibility of the fluid phase (*C*_f_) is
  (12) $$\begin{array}{c} C_{\text{f}}=C_{\text{w}}, \end{array}$$
  where *C*_w_ is water compressibility.
 (5) Compressibility of the matrix (*C*_m_) indicates the compressibility of sediments without water, and it can be calculated by the following equation:
  (13) $$\begin{array}{c} C_{\text{m}}=\left(1-\phi_{\text{eff}}\right)C_{\text{b}}+\phi_{\text{eff}}C_{\text{p}}, \end{array}$$
  where *C*_p_ is pore compressibility. The algorithm [35] to calculate *C*_p_ is:
  (14) $$\begin{array}{c} C_{\text{p}}=\frac{\left(1-\phi_{\text{eff}}/\phi_{0}\right)}{P_{\text{d}}}, \end{array}$$
  where *ϕ*_0_ is the sediment porosity at the sea bottom and *P*_d_ is differential pressure.
 (6) Proportional coefficient (*β*) is
  (15) β=CbCm.
 (7) Shear modulus (*μ*) indicates average rigidity of the skeleton, and it can be calculated by the following equation:
  (16) μ=(ϕs+ϕh)[ϕsSs′μsm+Sh′μh]−1,
  where *μ*_h_ is gas hydrate rigidity and *μ*_sm_ is the shear modulus of solid matrix with gas hydrate [55], which can be calculated by the following equation:
  (17) μsm=(μsmKT−μsm0)[ϕh(1−ϕs)]3.8+μsm0,
  where *μ*_smKT_ is Kuster and Toksoz's shear modulus [56] and *μ*_sm0_ is the shear modulus of solid matrix without gas hydrate.

#### 5.1.3. Implementation Steps of Numerical Simulation of Gas Hydrate Model Based on the TPT

Consider the following.

1. Given the *ϕ*, or calculate it by the empirical formula.
2. Given the *S*_h_ and *S*_w_, calculate *ϕ*_s_, *ϕ*_h_, *ϕ*_w_, *S*_s_′, and *S*_h_′ according to (2)–(6).
3. According to (7), calculate *ϕ*_eff_.
4. According to (8) and (10), calculate *ρ*_b_, *ρ*_f_, and *ρ*_m_.
5. According to (11)–(14), calculate *C*_b_, *C*_f_, and *C*_m_.
6. According to (15), calculate *β*.
7. According to (16) and (17), calculate *μ*.
8. According to (1), calculate *V*_p_.

### 5.2. Free Gas Model

#### 5.2.1. Establishment of Free Gas Model

Free gas model assumes that the sediments are composed of rock grain, water, and free gas, and it can be expressed as

(18) $$\begin{array}{c} \phi_{\text{s}}+\phi_{\text{w}}+\phi_{\text{g}}=1, \end{array}$$

(19) $$\begin{array}{c} \phi =\phi_{\text{w}}+\phi_{\text{g}}. \end{array}$$

Water saturation (*S*_w_) and free gas saturation (*S*_g_) can be expressed as

(20) Sw=ϕwϕ,

(21) Sg=ϕgϕ.

#### 5.2.2. The Parameter Determination for Numerical Simulation of Free Gas Model Based on the TPT

Consider the following.

  (1) *ϕ*_eff_ can be written as
  (22) ϕeff=ϕ.
 (2) *ρ*_m_, *ρ*_b_, and *ρ*_f_ can be written as
  (23) ρm=(1−ϕeff)ρb+ϕeffρf=(1−ϕ)ρb+ϕρf,
  (24) $$\begin{array}{c} \rho_{\text{b}}=\rho_{\text{s}}, \end{array}$$
  (25) $$\begin{array}{c} \rho_{\text{f}}=S_{\text{w}}\rho_{\text{w}}+S_{\text{g}}\rho_{\text{g}}, \end{array}$$
  where *ρ*_g_ is free gas density.
 (3) *C*_b_ can be written as
  (26) $$\begin{array}{c} C_{\text{b}}=C_{\text{s}}. \end{array}$$
 (4) Assume that the fluid compressibility lies between the Voigt and Reuss averages [54]. *C*_f_ can be written as
  (27) Cf=12(SwCw+SgCg)+12(SwCw+SgCg)−1,
  where *C*_g_ is the compressibility of free gas.
 (5) *C*_m_ is calculated by the same equation as that used in gas hydrate model.
  (6) *β* is calculated by the same equation as that used in gas hydrate model.
 (7) *μ* can be written as
  (28) μ=μs,  μs=μsm01−ϕ,
  where *μ*_*s*_ is rock grain rigidity.
 (8) Variation range of *k* is 1–∞.

#### 5.2.3. Implementation Steps of Numerical Simulation of Free Gas Model Based on the TPT

Consider the following.

1. Given the *ϕ*, or calculate it by the empirical formula.
2. Given the *S*_g_ and *S*_w_, calculate *ϕ*_s_, *ϕ*_w_, and *ϕ*_g_ according to (18)–(21).
3. According to (22), calculate *ϕ*_eff_.
4. According to (23) and (25), calculate *ρ*_b_, *ρ*_f_ and *ρ*_m_.
5. According to (11)–(14), calculate *C*_b_, *C*_f_, and *C*_m_.
6. According to (15), calculate *β*.
7. According to (16) and (17), calculate *μ*.
8. According to (1), calculate *V*_p_.

### 5.3. Estimation of Sediment Porosity

Sediment porosity is a key parameter for the estimation of gas hydrate saturation for both gas hydrate and free gas models. Therefore, appropriate log data should be selected to estimate the sediment porosity at site SH2. The log data that can be applied to determine the sediment porosity include density, acoustic, resistivity, and neutron logging.

When it comes to the determination of sediment porosity by acoustic log, the data need to be corrected by the regional core data because seafloor sediments are always loose silt and silty clay. However, the core data is always insufficient, and it is difficult to determine the compaction correction coefficient, so acoustic log is not available. When it comes to the determination of sediment porosity by resistivity log, the Archie formula should be used to calculate porosity, and the Archie constants and formation water resistivity need to be known for the Archie formula [57]. However, the previous two parameters are generally determined by some empirical equations, and the estimation error of the sediment porosity is significant. As for the determination of sediment porosity by neutron log, it usually cannot be realized because of the lack of the neutron log data.

Compared with the resistivity, the acoustic and the density logs are less affected in gas hydrate reservoirs, and can generally reflect the situation of sediment porosity, so we select the density log data to estimate the sediment porosity in this study. The intensity of scattering gamma ray can be measured by the density log, which reflects electron density of the strata and volume density of rock (*ρ*_b_). The estimation of *ϕ* by density log data can be expressed as [58]

(29) ϕ=(ρma−ρb)(ρma−ρf),

where *ρ*_ma_ is matrix density and *ρ*_f_ is fluid density. Considering the effect of shaly sediments, (29) can be written as [58]

(30) ϕ=(ρma−ρb)(ρma−ρf)−Vsh(ρma−ρsh)(ρma−ρf),

(31) Vsh=(2GCUR×SH−1)(2GCUR−1),

(32) $$\begin{array}{c} \text{SH}=\frac{\left(\text{GR}-\text{G}{\text{R}}_{\min}\right)}{\left(\text{G}{\text{R}}_{\max}-\text{G}{\text{R}}_{\min}\right)}, \end{array}$$

where *V*_sh_ is the volume content of the shale, SH is the content index of the shale, *ρ*_sh_ is the density of the shale, GR is the value of natural gamma log in the research interval, GR_min⁡_ is the value of natural gamma log in pure sandstone interval, GR_max⁡_ the is value of natural gamma log in pure mud interval, and GCUR is the Hilchie index, which is 3.7 in the Tertiary of North America and 2 in old stratum [59].

**Figure 3:**
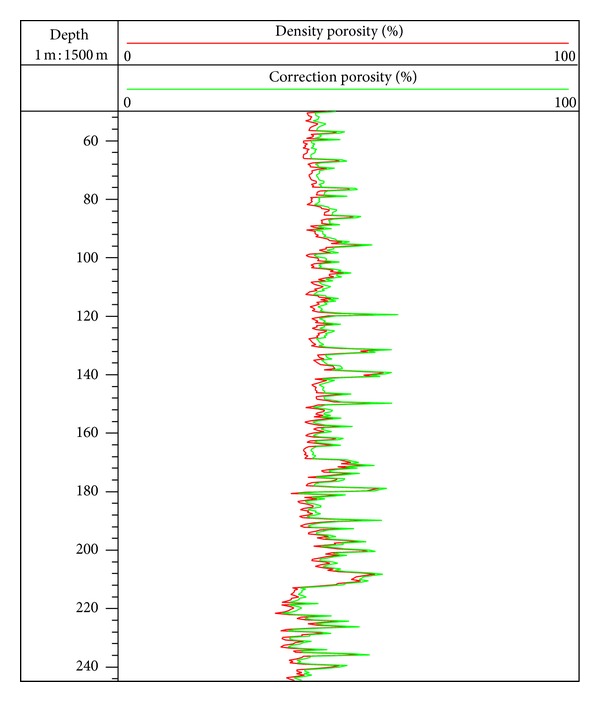
Result of sediment porosity calculated by density log data at site SH2. (Red line is the sediment porosity estimation used density log data; bright green line is the sediment porosity estimation which considered the effect of argillaceous sediments.)

Equations (29) and (30) are used to calculate the sediment porosity at site SH2, and processing parameters can be set as follows: *ρ*_ma_ = 2.65 g/cm^3^, *ρ*_f_ = 1.04 g/cm^3^, *ρ*_sh_ = 2.70 g/cm^3^ [12, 35]. The porosity calculated by (29) and (30) is close to each other (Figure 3), which varies in the range of 30% to 55%, and the average value is 45%. The result indicates that sediments at site SH2 are of high porosity.

## 6. Results 

**Figure 4:**
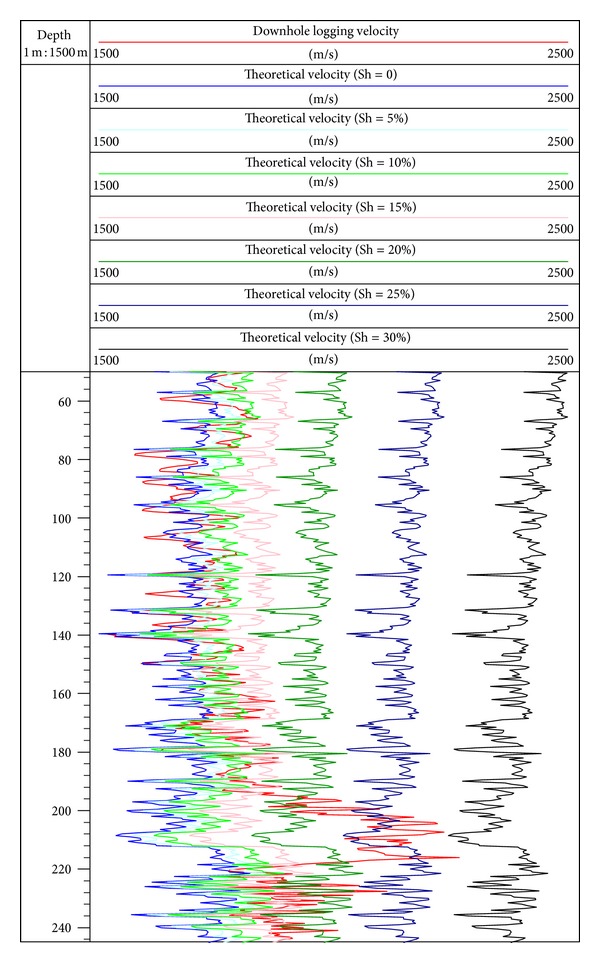
Forward stimulating P wave velocity of sediment formation at site SH2. (The red line is the actual log P wave velocity at site SH2; the blue line, sky blue line, bright green line, pink line, green line, dark blue line, and black line are assumed to P wave velocity made by forward modeling of the gas hydrate model when gas hydrate saturations are 0, 5%, 10%, 15%, 20%, 25%, and 30%, resp.)

Gas hydrate model of the TPT is used to forward stimulate P wave velocity of sediment formation in different gas hydrate saturation conditions in depth of 50 to 245 mbsf at site SH2.  Table 1 shows the values of the main parameters used to evaluate the velocity. When S_h_ = 0, we can get the P wave velocity of water saturated sediments forward stimulated by gas hydrate model based on the TPT. From Figure 4, the tendency between actual curve of P wave velocity log and P wave velocity curve of the saturated water condition is almost consistent in the gas hydrate interval (above 195 mbsf), so the model and its parameters are rational for numerical simulation in this study. The difference between actual P wave velocity of the log and P wave velocity of saturated water condition reflects the value of gas hydrate or free gas saturation, which can be used to qualitatively identify the gas hydrate reservoir. The specific response characteristics are as follows: the possibility of containing gas hydrate is dominant when the actual P wave velocity of the log is higher than P wave velocity of saturated water condition; the possibility of containing free gas is dominant when actual P wave velocity of the log is lower than the P wave velocity of saturated water condition [35]. In depth of 195 to 220 mbsf, actual P wave velocity of the log is significantly higher than the P wave velocity of saturated water condition, so this interval is the gas hydrate stability zone. In depth of 220 to 245 mbsf, the actual P wave velocity of the log has an increase relative to the P wave velocity of saturated water condition. However, Figure 2 does not indicate the increase of resistivity. Without the coring analysis data in this interval, whether the abnormality is caused by gas hydrate or not can not be ascertained, and should be researched in further study. 

When S_h_ gradually increases, P wave velocity made by forward stimulation also increases; when S_h_ > 15%, P wave velocity increases significantly; when S_h_ = 30%, P wave velocity curve is located at the right of the actual P wave velocity logging curve. The above results indicate that the basic range of gas hydrate saturation is 0–30% in depth of 50 to 245 mbsf at site SH2.

**Figure 5:**
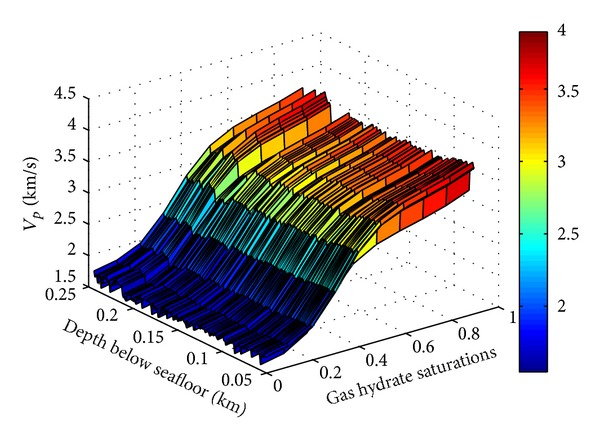
Relation between P wave velocity and sediment porosity, gas hydrate saturation at site SH2. (Because of the effect of variation of borehole conditions and actual sediments, the sediment porosity in some intervals does not reduce with the increasing depth and causes the curved surface unsmooth growth. In depth of 195 to 220 mbsf of gas hydrate reservoir, P wave velocity surface of forward simulation subsides as the sediment porosity relatively increases.)

In order to study the dependence of P wave velocity on sediment porosity, gas hydrate saturation, it is assumed that values of gas hydrate saturation increase from 0 to 1 in the interval of 0.1. Using gas hydrate model of the TPT to model the corresponding P wave velocity of the previous gas hydrate saturations, the relation surface of previous three properties can be formed as Figure 5 shows. With the increase of the sediment burial depth in depth of 50 to 245 mbsf at site SH2, the porosity presents a decreasing trend except for the abnormality caused by borehole conditions in some intervals, and P wave velocity of forward stimulation (S_h_ = 0) slowly increases from 1743 to 1795 m/s. But with the increase of gas hydrate saturation, the increase rate of P wave velocity is obviously accelerated, and P wave velocity (burial depth is 51 mbsf) increases from 1743 to 3961 m/s. From the above analysis, the general rule between P wave velocity of forward stimulation and sediment porosity, gas hydrate saturation at site SH2 is the smaller the sediment porosity, the greater the P wave velocity; the higher the gas hydrate saturation, the greater the P wave velocity. This result is basically in accordance with the research result made by Tinivella [35].

**Figure 6:**
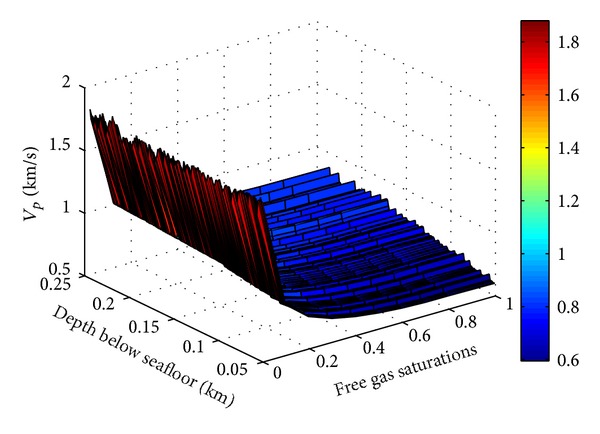
Relation between P wave velocity and sediment porosity, free gas saturation at site SH2. (P wave velocity is easily affected by the free gas saturation. When the free gas saturation increases, P wave velocity of forward stimulation by the free gas model of the TPT decreases rapidly.)

Similarly, it is assumed that values of free gas saturation increase from 0 to 1 in the interval of 0.1 in order to study the relation between P wave velocity and free gas saturation. Using free gas model of the TPT to model the corresponding P wave velocity of the previous free gas saturations, the relation surface of P wave velocity, sediment porosity, and free gas saturation can be formed as Figure 6 shows. In depth of 50 to 245 mbsf at site SH2, with the increase of free gas saturation, P wave velocity (burial depth is 51 mbsf) decreases from 1773 to 597 m/s (The velocity 597 m/s is obtained supposing 100% free gas saturation), and the decrease rate is significant. Considering the depth effect, the decrease rate of P wave velocity slows down with the increase of burial depth. From the above analysis, the general rule between P wave velocity of forward stimulation and free gas saturation at site SH2 is the higher the free gas saturation, the lower the P wave velocity. This result is also basically in accordance with the research result made by Tinivella [35].

The estimation of gas hydrate saturation for gas hydrate reservoir evaluation has an important significance. In order to estimate gas hydrate saturation in sediments, it is necessary to associate the P wave velocity of the log with the P wave velocity of gas hydrate model based on the TPT. Given an initial gas hydrate saturation, based on gas hydrate model of the TPT, the difference between P wave velocity of forward simulation and the actual P wave velocity of the log in this saturation can be acquired. If the difference is in the range of allowable error, the saturation can be treated as the actual saturation; if the difference does not satisfy the error requirement, the value of gas hydrate saturation should be modified until meeting the error precision.

**Figure 7:**
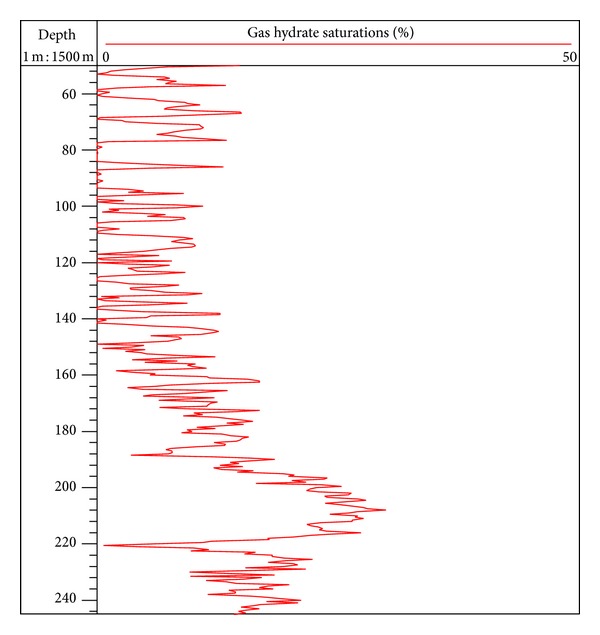
Estimation of gas hydrate saturation at site SH2.

Using gas hydrate model of the TPT to inverse gas hydrate saturation at site SH2, the values of the main parameters are listed in Table 1, and the inversion result is shown in Figure 7. In the interval of 50 to 90 mbsf at site SH2, the range of gas hydrate saturation is 0–17.5%, and the average value is 4.8%. As the shallow sediments are influenced by variation of borehole conditions, the estimation error of gas hydrate saturation in this interval is significant, which should be noticed during the analysis. In the interval of 90 to 195 mbsf, the range of gas hydrate saturation is 0–18.9%, and the average value is 7%. In the interval of 195 to 220 mbsf, the range of gas hydrate saturation is 7–31.5%, and the average value is 23.2%. With the increase of burial depth, gas hydrate saturation gradually increases and finally reaches the peak value of 31.5% in 208 mbsf. Then the gas hydrate saturation decreases slowly with the increase of burial depth, and the range of gas hydrate saturation is 0–25.8% in the interval of 220 to 245 mbsf, with an average value of 15.5%.

## 7. Discussion

**Figure 8:**
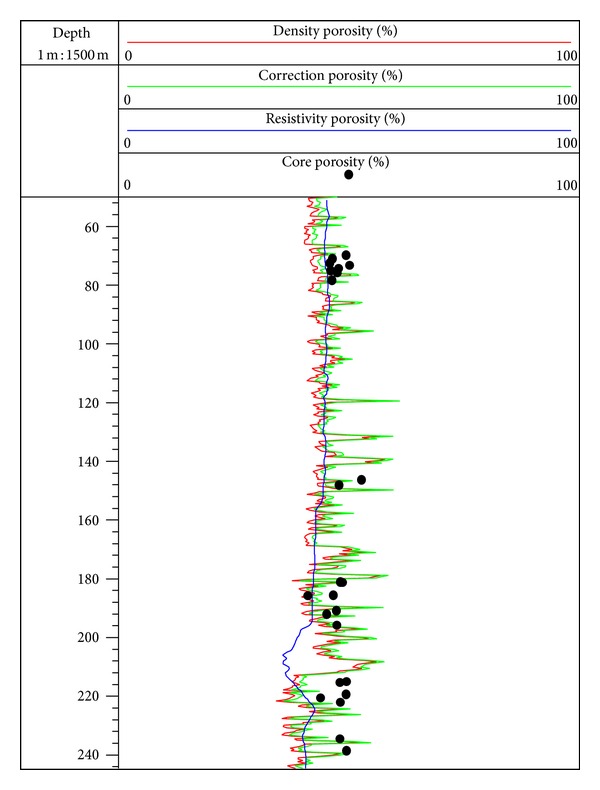
Comparison of estimation of sediment porosity made by different methods at site SH2. (The red curve is the porosity estimated by density log data; the bright green curve is the porosity considering the effect of shaly sediments; blue curve is the porosity estimated by resistivity log combined with Archie formula; the black dots are the porosity measured by core laboratory.)

It is very important to determine the porosity for the evaluation of gas hydrate saturation. In order to analyze the accuracy of porosity estimation by density log data, we use resistivity log data and combine the Archie formula [57] to make a comparison between the estimation results, and the comparison results are shown in Figure 8. The porosity estimated by resistivity log data generally changes in the range of 30 to 50%, and the average value is 43% [12, 64]. In the interval of 50 to 195 mbsf, the curves of density porosity and resistivity porosity are approximately coincident, while the former fluctuates due to the borehole effect. In the interval of 195 to 220 mbsf, the sediment contains gas hydrate, and the curve of resistivity porosity decreases significantly compared with the curve of density porosity due to the significant increase of resistivity (Figure 2), so the porosity calculated by resistivity log data needs to be corrected to exclude the influence of the increase of skeleton components. In the interval of 220 to 245 mbsf, the curves of density porosity and resistivity porosity are approximately coincident again. These results indicate that using density log data to estimate the porosity in the gas hydrate stability zone at site SH2 is relatively more reliable. According to previous studies, the range of core porosity by laboratory analysis in this interval was 40–55%, which was almost coincident with the estimation of porosity by density log data in this study [13]. As Figure 8 shows, the core porosity distribution corresponds with the curve of density porosity of the well, and this result proves that the porosity estimated by density log data can meet the requirement for evaluating gas hydrate saturation at site SH2.

**Figure 9:**
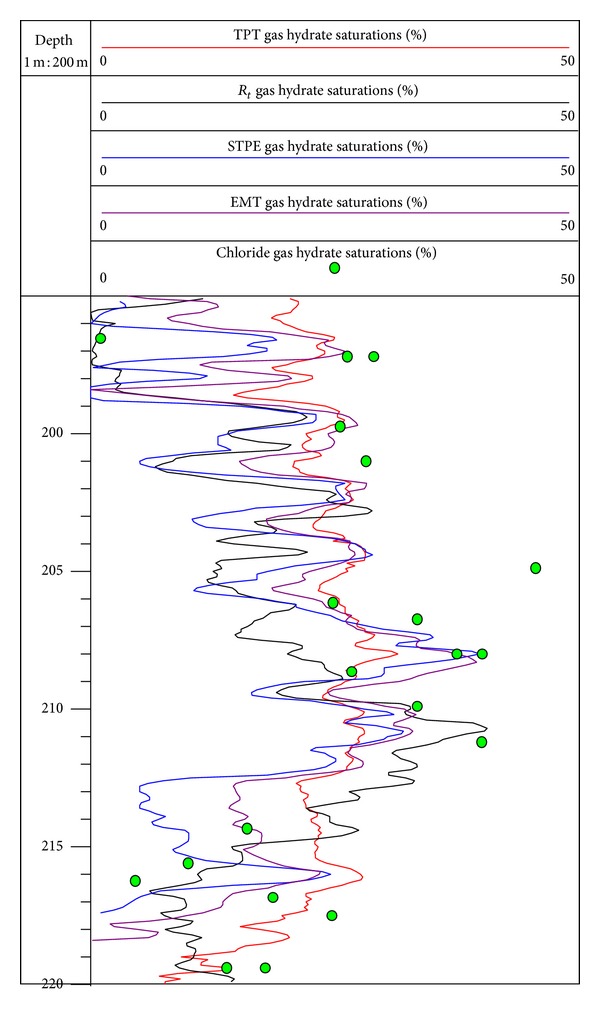
Comparison of estimation of gas hydrate saturation made by different methods in depth of 195 to 220 mbsf at site SH2. (The red curve, black curve, blue curve, and violet curve represent gas hydrate saturations estimated by the TPT, the Rt, the STPE, and the EMT method, respectively; the bright green dots represent gas hydrate saturations calculated by chloride anomaly method.)

In order to verify the accuracy of gas hydrate saturation estimated by the TPT, we compare gas hydrate saturation in this study with that estimated by Wang et al. [50] in the occurrence of gas hydrate (195 to 220 mbsf) at site SH2 (Figure 9). The curve of gas hydrate saturation made by the TPT first increases and then decreases as the burial depth increases, which reaches the peak value of 31.5% in the 208 mbsfs and then decreases gradually. The peak value of gas hydrate saturation by the TPT is slightly smaller than the resistivity log (Rt) method (40.5%), the simplified three-phase equation (STPE) method (41%), and the effective medium theory (EMT) method (38.5%). However, the curve trend of gas hydrate saturation estimated by the TPT and other three methods is basically consistent, and the difference only lies in the amplitude of the curve, which indicates that using the TPT method to estimate gas hydrate saturation at site SH2 is available.

The average value of gas hydrate saturations calculated by chloride anomaly method is 25%, and the peak value is 45% [9]. The peak value of gas hydrate saturation estimated by the TPT is relatively lower than that estimated by chloride anomaly method, but the average values estimated by the two methods are basically same, and most distribution dots of gas hydrate saturations obtained by chloride anomaly method correspond with the curve of gas hydrate saturation estimated by the TPT method (Figure 9). This also indicates that using the TPT method to estimate gas hydrate saturation at site SH2 is available.

## 8. Conclusions

In summary, the relationships between P wave velocity and gas hydrate saturation, free gas saturation, and sediment porosity at site SH2 are studie, respectively, by virtue of elastic wave velocity of numerical stimulation based on the TPT, and gas hydrate model and free gas model are established to estimate the sediment porosity in order to determine the gas hydrate saturation of the research area. Some conclusions can be drawn as follows.

(1) Using the difference between P wave velocity of saturated water condition and actual P wave velocity of the log, whether the sediment contains gas hydrate or not can be identified quickly. In the interval of 195 to 220 mbsf at site SH2, the actual P wave velocity of the log increases significantly relative to P wave velocity of forward stimulation in saturated water condition, so this interval is determined to contain gas hydrate.

(2) By virtue of elastic wave velocity of numerical stimulation based on the TPT, combined with log data, the dependence of P wave velocity on gas hydrate saturation, free gas saturation, and sediment porosity at site SH2 can be analyzed, respectively. In the interval of 50 to 245 mbsf, as sediment porosity decreases, P wave velocity gradually increases; as gas hydrate saturation increases, P wave velocity increases; as free gas saturation increases, P wave velocity gradually decreases.

(3) The log data can be used to calculate gas hydrate saturation of the whole well, and the availability is better than the coring data. The average value of gas hydrate saturation estimated by the TPT is 23.2%, and the peak value is 31.5%, which is basically in accordance with the values estimated by the STPE model, the EMT model, the Rt model and chloride anomaly method.

## Figures and Tables

**Table 1:** Values of the main parameters of the gas hydrate and free gas model of the TPT.

Parameters	Details	References
*C*_s_	2.7 × 10^−11^ Pa^−1^	[60]
*C*_h_	1.79 × 10^−10^ Pa^−1^	[1]
*C*_w_	4.79 × 10^−10^ Pa^−1^	[61]
*C*_g_	4.24 × 10^−8^ Pa^−1^	[62]
*ρ*_s_	2650 kg/m^3^	[60]
*ρ*_w_	1040 kg/m^3^	[1]
*ρ*_g_	88.48 kg/m^3^	[62]
*ρ*_h_	767 kg/m^3^	[1]
*V*_s_	116 + 4.65*z* m/s 237 + 1.28*z* m/s 332 + 0.58*z* m/s	[63]
*μ*_sm0_	*ρ*_m_ *V*_s_^2^ Pa	[35]
*μ*_h_	3.7 × 10^9^ Pa	[35]
*k*	2.3	[61]
